# Amygdalar Endothelin-1 Regulates Pyramidal Neuron Excitability and Affects Anxiety

**DOI:** 10.1038/s41598-017-02583-6

**Published:** 2017-05-24

**Authors:** Ming Chen, Huan-huan Yan, Shu Shu, Lei Pei, Long-kai Zang, Yu Fu, Ze-fen Wang, Qi Wan, Lin-lin Bi

**Affiliations:** 1 Wuhan University Center for Pathology and Molecular Diagnostics, Zhongnan Hospital of Wuhan University, Wuhan, 430071 China; 2 Department of Pathology, Wuhan University School of Basic Medical Sciences, Wuhan, 430071 China; 3 Department of Cardiology, Zhongnan Hospital of Wuhan University, Wuhan, 430071 China; 4 Department of Pathophysiology and Key Laboratory of Neurological Diseases of Ministry of Education, Tongji Medical College, Huazhong University of Science and Technology, Wuhan, 430030 China; 5 Department of Physiology, Wuhan University School of Basic Medical Sciences, Wuhan, 430071 China

## Abstract

An abnormal neuronal activity in the amygdala is involved in the pathogenesis of anxiety disorders. However, little is known about the mechanisms. High-anxiety mice and low-anxiety mice, representing the innate extremes of anxiety-related behaviors, were first grouped according to their anxiety levels in the elevated plus maze test. We found that the mRNA for endothelin-1 (ET1) and ET1 B-type receptors (ETBRs) in the amygdala was down-regulated in high-anxiety mice compared with low-anxiety mice. Knocking down basolateral amygdala (BLA) ET1 expression enhanced anxiety-like behaviors, whereas over-expressing ETBRs, but not A-type receptors (ETARs), had an anxiolytic effect. The combined down-regulation of ETBR and ET1 had no additional anxiogenic effect compared to knocking down the ETBR gene alone, suggesting that BLA ET1 acts through ETBRs to regulate anxiety-like behaviors. To explore the mechanism underlying this phenomenon further, we verified that most of the ET1 and the ET1 receptors in the BLA were expressed in pyramidal neurons. The ET1–ETBR signaling pathway decreased the firing frequencies and threshold currents for the action potentials of BLA pyramidal neurons but did not alter BLA synaptic neurotransmission. Together, these results indicate that amygdalar ET1-ETBR signaling could attenuate anxiety-like behaviors by directly decreasing the excitability of glutamatergic neurons.

## Introduction

Despite the high prevalence of anxiety disorders^1, 2^, the neural circuitry underlying such disorders has not been fully clarified. Available treatments, such as classical 1,4-benzodiazepines acting on the GABA_A_ receptor/chloride channel, are inconsistently effective and addictive. Moreover, these drugs have muscle relaxant, cognitive impairment and respiratory suppression effects^3, 4^. These findings highlight the need for a deeper understanding of anxiety control mechanisms in the central nervous system (CNS).

The amygdala, which is composed of functionally and morphologically heterogeneous subnuclei with complex interconnectivity, is very important in modulating anxiety-related responses^5, 6^. The basolateral amygdala (BLA) is primarily composed of glutamatergic neurons (~90%)^7, 8^, whereas the central nucleus of the amygdala (CeA) consists of ~95% GABAergic neurons^9^. The BLA receives sensory inputs from the cortex and thalamus and relays information to the CeA, which contains projection neurons that innervate neurons in the brain stem and the hypothalamus^6, 10–12^. Patients with anxiety disorder might exhibit abnormal neuronal activity in the BLA, particularly, abnormal glutamatergic activity in the BLA^12–14^. However, the mechanisms regulating glutamatergic activity in the amygdala in relation to anxiety have not been extensively studied. Because the BLA serves as the gateway to the amygdala and controls the fear information transmitted to the CeA, we aimed to explore the function of the BLA in controlling anxiety.

Endothelin-1 (ET1) is a 21-amino-acid peptide and a potent vasoconstrictor that was first isolated from the supernatant of cultured porcine aortic endothelial cells^15^. Although many previous human studies have shown links between ET1 and diseases such as arterial hypertension and renal disease^16–19^, recent patient data has shown possible connections between ET1 and anxiety^20, 21^ and between ET1 and depression^22^. ET1 is widely distributed in the CNS and acts on two distinct G protein-coupled receptors: the ETA receptor (ETAR) and the ETB receptor (ETBR) subtypes^16, 23–25^. Previous studies have shown that heterozygous ET1-knockout mice exhibit different behavioral responses to stressors and that transgenic mice over-expressing ET1 in endothelial cells show increased anxiety-like behaviors in the open-field test^24, 26^. Our previous work found that exogenous administration of ET1 into the infralimbic cortex induced anxiety-like behaviors due to its direct synaptic neurotransmission properties^21^. Additionally, another report showed that unilateral infusion of ET1 into the medial prefrontal cortex (mPFC) resulted in a pronounced and persistent anxiety and depression phenotype with no evident sensorimotor deficits^27^. However, the cell type-specific distribution and function of ET1 in other brain regions (especially in the amygdala, which is a key brain area for anxiety) and the direct neuronal excitability mechanism mediated by ET1 in the regulation of anxiety remain unclear.

Here, we investigated whether ET1–ETBR signaling in the amygdala modulates anxiety-like behaviors. Furthermore, we evaluated whether ET1–ETBR regulates the excitability of GABAergic and/or glutamatergic neurons in the amygdala. We used the elevated plus maze test to discriminate innate extremes in anxiety-related behaviors, and we then examined candidate genes (ET1, ETBR and ETAR) for involvement in anxiety-related behaviors. We found that high-anxiety mice expressed low levels of ET1 and ETBR in the amygdala. ET1 and its receptors are mostly expressed in BLA pyramidal neurons. ET1, through its receptor ETBR, regulates the excitability of BLA pyramidal neurons and anxiety-like behaviors. Up-regulating ETBR gene expression in the BLA by using ETBR lentiviral activation particles (LV-ETBR) attenuated anxiety-related behaviors and concomitantly inhibited the excitability of BLA pyramidal neurons. These observations reveal a novel function of ET1-ETBR in the amygdala, identify a novel pathophysiological mechanism, and may suggest a target for the development of a new class of anxiolytic drugs.

## Methods and Materials

### Subjects

Adult male C57 BL/6 mice aged 10–12 weeks and weighing 20–25 g at the time of testing were housed (four to five per cage) in standard laboratory cages on a 12-h light/dark cycle (lights on at 8:00 A.M.) in a temperature-controlled room (21–25 °C). The mice were kept with free access to food and water. Behavioral testing was performed during the light cycle between 10:00 A.M. and 4:00 P.M. Procedures were in accordance with the Chinese Council on Animal Care Guidelines^28^. All experiment protocols were also conducted in accordance with guidelines set by the Wuhan University and approved by the Committee on Animal Care and Use of the Basic Medical Sciences at the Wuhan University. Efforts were made to minimize mouse suffering and to reduce the number of mice used.

### Drugs

ET1 shRNA Lentiviral Particles (sc-45395-v), ETAR Lentiviral Activation Particles (sc-420111-LAC), ETBR Lentiviral Activation Particles (sc-420112-LAC), ETBR shRNA (m) Lentiviral Particles (sc-39963-V) and ETAR shRNA (m) Lentiviral Particles (sc-39961-V) were bought from Santa Cruz Biotech. The control Lentiviral Particles (LV-GFP) were bought form Neuron Biotech.

Bicuculline methiodide (BMI; Tocris Bioscience, UK) was dissolved in dimethyl sulfoxide (DMSO), and the final concentration of DMSO was less than 0.1%. D (−)-2-Amino-5- phosphonopentanoic-acid (AP5; Sigma-Aldrich, USA) and 6- cyano-7-nitroquinoxaline-2,3-dione (CNQX; Tocris Bioscience, UK) were dissolved in artificial cerebrospinal fluid (ACSF). All other chemicals were from Sigma-Aldrich. Dose selections for these drugs were based on previously published studies^29–33^.

### Quantitative Real-Time PCR

As previously reported^34, 35^, mouse brain samples were dissected and prepared after selection. To make preparations for the PCR, we dissected the mice brain 24 h after the anxiety test. The cortex (mainly from piriform cortex neighboring the BLA, −1.5 mm posterior to the bregma) and amygdala (mainly from BLA or mainly from CeA) were separated from frozen sections. Different brain tissue was placed immediately in Trizol (Invitrogen), and RNA was extracted according to the manufacturer’s manual. Genomic DNA was removed by gDNA eraser treatment (Takara), and 1 mg RNA was used for first-strand cDNA synthesis (Takara). For real-time RT-qPCR, an SYBR detection system (Takara), specific primers (ET1-F: 5′-TGCTGTTCGTGACTTTCC-3′ and ET1-R: 5′-TGTTGACCCAGATGATGTC-3′; ETAR-F:5′-GCTGGTTCCCTCTTCACTTAAGC-3′; ETAR-R:3′-TCATGGTTGCCAGGTTAATGC-5′; ETBR-F:5′-AAGATTGGTGGCTGTTCAGTTTCT-3′; ETBR-R:3′-GAGCATTTCGCAGGTCATCA-5′), and 2 ml of undiluted cDNA were used in 20 ml PCR reactions. Each reaction was performed in duplicate. All real-time RT-PCR reactions were performed in 40 cycles on the iCycler (Agilent Technologies Stratagene Mx3005 P). The relative gene expression and statistical analysis were determined using the Relative Expression Software Tool.

### Immunostaining

Immunofluorescence staining was carried out as described previously^36^. Briefly, mice were transcardially perfused with 4% paraformaldehyde (4 g/100 ml) and 4% sucrose (4 g/100 ml) in PBS, pH 7.4. Brain tissue was removed and post-fixed at 4 °C for 24 h. Amygdala slices (30 μm) were prepared using a freezing microtome. Brain sections were treated with 3% (vol/vol) normal goat serum in PBS containing 0.5% Triton X-100 for 1 h. Then, the brain sections were incubated with primary antibody at 4 °C for 48 h. Slices were incubated with secondary antibodies for 2 h and exposed to 4′,6-diamidin-2-phenylindol (DAPI, 1:10000) for 5 min as a counterstain. The sections were examined with a laser-scanning confocal microscope (LSM 510, Carl Zeiss) using an omnichrome air-cooled helium/neon laser tuned to produce beams at 488 and 594 nm.

Primary antibodies used were anti-CaMKII (dilution 1:100, sc-5306, Santa Cruz Biotechnology), anti-GAD67 (dilution 1:250, MAB5406, Millipore), anti-ET1 (dilution 1:100, sc-21625, Santa Cruz Biotechnology), anti-ETAR (dilution 1:100, sc-33536, Santa Cruz Biotechnology) and anti-ETBR (dilution 1:100, sc-33538, Santa Cruz Biotechnology). All secondary antibodies were chosen according to the primary antibodies from Invitrogen. Cells were counted and analyzed by an experimenter who was blind to the sample.

### Electrophysiology

Slice preparation: the slices (350 μm) of the amygdala were prepared from male mice using a Vibroslice (Leica VT 1000 S) in an ice-cold solution that contained 220 mM sucrose, 2.5 mM KCl, 1.3 mM CaCl2, 2.5 mM MgSO4, 1 mM NaH2PO4, 26 mM NaHCO3, and 10 mM glucose. Slices were allowed to recover for at least 1.5 h (0.5 h at 34 °C followed by 1 h at 25 ± 1 °C) in an ACSF solution containing 126 mM NaCl, 26 mM NaHCO3, 3.0 mM KCl, 1.2 mM NaH2PO4, 2.0 mM CaCl2, 1.0 mM MgSO4, and 10 mM glucose. A single slice was then transferred to the recording chamber and submerged and perfused with ACSF (2 ml/min). All of the solutions were saturated with 95% O2/5% CO2.

Neurons were visualized with an infrared-sensitive CCD camera with a ×40 water-immersion lens (Zeiss, Axioskop2 Fsplus) and recorded using whole-cell techniques (MultiClamp 700B Amplifier, Digidata 1320 A analog-to-digital converter) and pClamp 9.2 software (Axon Instruments). For action potential recording, glass pipettes (3–5 MΩ) were filled with a solution containing 140 mM potassium gluconate, 2 mM NaCl, 10 mM HEPES buffer, 2 mM Mg-ATP, 0.3 mM Na-GTP, and 0.2 mM EGTA (pH 7.2 with KOH, 285 mOsm). The threshold current for spike generation was the minimum depolarizing current needed to elicit at least one action potential. A series of current steps (500 ms duration, 0 to 500 pA range with 100 pA step increments) were injected into the cell with I-clamp until an action potential was generated.

According to our previous study^5^, miniature excitatory postsynaptic currents (mEPSCs) were recorded with 1 μM TTX and a V-clamp in the presence of the GABA_A_R antagonist, BMI (20 μM). To record mEPSCs, glass pipettes were filled with the following solution: 105 mM K-gluconate, 30 mM KCl, 10 mM HEPES, 10 mM phosphocreatine, 4 mM ATP-Mg, 0.3 mM GTPNa0.3 mM EGTA, and 5 mM QX314 (pH 7.35, 285 mOsm). Miniature inhibitory postsynaptic currents (mIPSCs) were recorded in the presence of AP5 (50 μM), CNQX (20 μM) and 1 μM TTX. To record mIPSCs, pipettes were filled with the following solution: 140 mM CsCl, 10 mM HEPES, 0.2 mM EGTA, 1 mM MgCl2, 4 mM Mg-ATP, 0.3 mM Na-GTP, and 5 mM QX314 (pH 7.25, 285 mOsm). The resistance of the pipettes was 3–5 MΩ. The holding potential for mEPSCs and mIPSCs was −70 mV. Data were collected when series resistance fluctuations remained within 15% of the initial value (10–15 MΩ). Data were filtered at 2 kHz and were sampled at 10 kHz.

### Surgery and Viral Injection

Viral vectors were injected into the BLA as described^5^. Mice were anesthetized and placed in a stereotaxic frame (Stoelting, USA). The mouse scalp was removed, and small burr holes were drilled into the skull (1 mm diameter) with a drill. Virus (0.5 μl) was infused using a 10 μl Hamilton syringe with a 33 gauge blunt tipped needle and a microinjector pump at a rate of 0.2 μl/min; the needle rested in position for 5 min post-injection. The injection coordinates were −1.5 mm posterior to the bregma, ±3.2 mm lateral to the midline, and −4.7 mm the from the pia surface. Following injection, incisions were sutured and topical anesthetic was applied to the wound (Bupivicaine, 0.5%). Mice recovered for 14 d before behavioral analysis.

### Behavioral Tests

#### Open Field Test

As previously described^5^, the open field testing chamber composed of gray polyvinyl chloride was a rectangular chamber (60 × 60 × 40 cm). The center area was illuminated by halogen bulbs (200 lux, 200 cm above the field). Mice were gently placed into one corner of the testing chamber and were allowed 5 min of free movement, which was monitored by an automated video tracking system. Images of the activities in those 5 min were automatically analyzed using the DigBehv animal behavior analysis program.

#### Elevated plus maze (EPM) test

As with our previous studies^5^, the test consists of an elevated, plus-sign-shaped runway that was ~40 cm above the floor, with two opposing closed arms (10 × 50 × 40 cm) and two open arms (10 × 50 cm) and one intersection (10 × 10 cm). Mice were allowed to acclimate to the testing room 30 min before the test. At the time of the test, each mouse was placed at the intersection center of the EPM, facing the closed arm and was videotaped for 5 min. The time spent in the closed and open arms was quantified autonomously by the DigBehv animal behavior analysis software.

#### Novelty-Suppressed Feeding Test

According to our previous work^5^, after 24 h of food (but not water) deprivation, mice were placed into the testing box. The box floor was covered with 2-cm-thick padding, and one single pellet of food was placed on a white piece of paper positioned at the center of the testing box (50 × 50 × 20 cm). A stopwatch was used to measure 5 min time. Latency was scored as the time at which the mice began biting the food. If mice did not bite the food in 5 min, the latency was scored as 5 min. Immediately after that, the mice were transferred to their home cage for another 5 min, and the amount of food intake over this time was measured (home cage food intake).

### Statistical Analyses

The number of experimental animals is indicated by “n”. All the data met the assumption of normality. Data were analyzed by student’s t test, one-way or two-way ANOVA. For multiple comparison, SNK test was used when equal variances assumed, Dunnett’s test was used when equal variances were not assumed. For paired comparison, two-way ANOVA was followed by repeated measures ANOVA. Throughout the study, statistical analyses were performed using SPSS software (SPSS, Inc.). All data are expressed as the mean ± SEM. Values of p < 0.05 were considered significant.

## Results

### ET1 mRNA levels in the amygdala of high-anxiety mice are lower than those of low-anxiety mice

**Figure 1:**
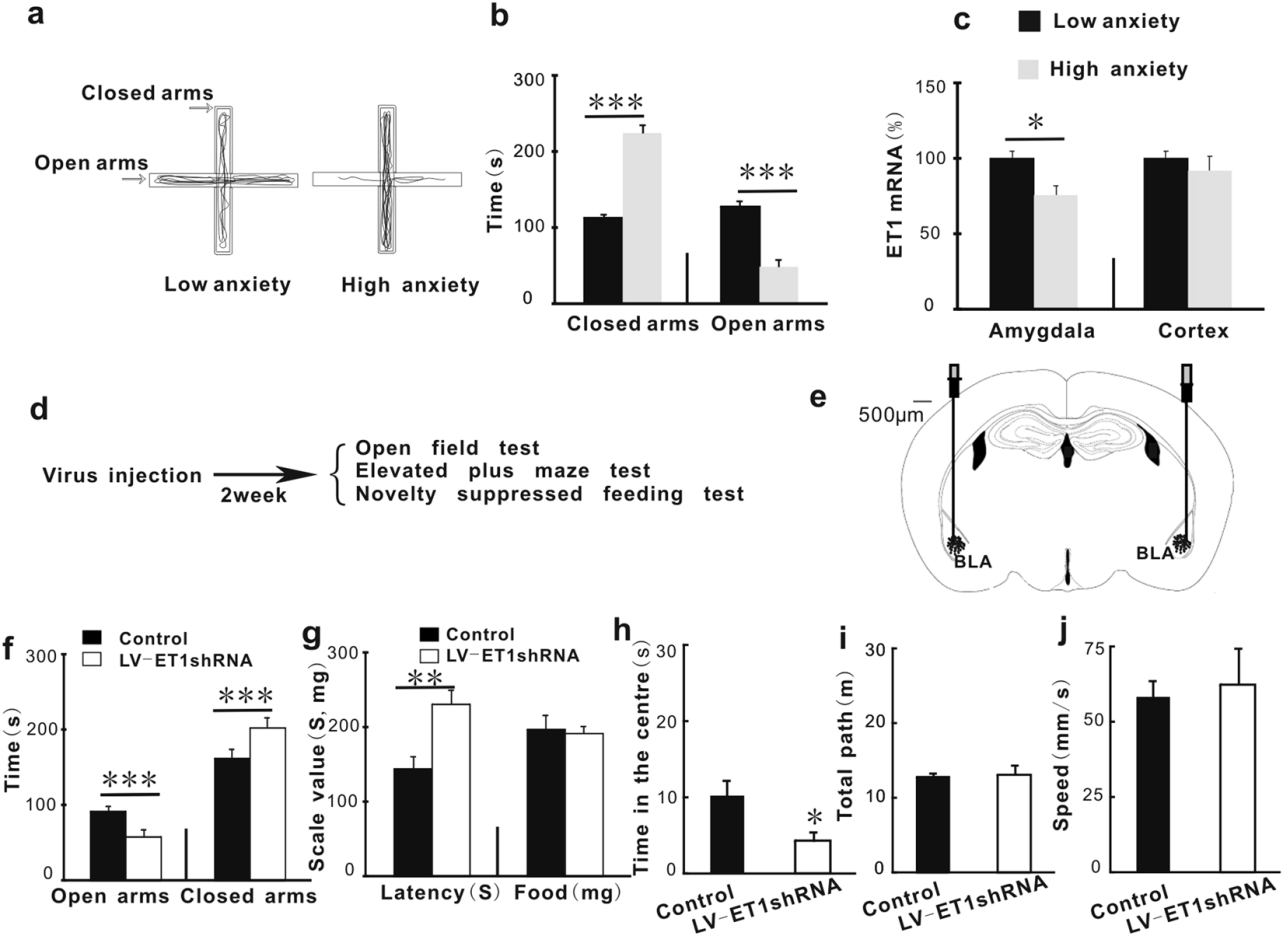
ET1 mRNA levels in the amygdala of high-anxiety mice are lower than in low-anxiety mice, and infusing ET1 gene knock-down lentiviral vector (LV-ET1 shRNA) into the BLA enhanced anxiety-like behaviors. (**a**) Schematic illustration of the elevated plus maze test. The left trace is the path of a representative low-anxiety mouse, and the right trace is that of a high-anxiety mouse. This system is an acute selection of anxiety-like behavior and not a model of the trait anxiety. (**b**) The time spent in the open arms and the time spent in the closed arms in the elevated plus maze test. (**c**) The real-time quantitative fluorescence PCR analysis shows ET1 mRNA expression (n = 5/group). (**d**) The experimental protocol. (**e**) Infusion sites in the BLA. The infusion sites of the tips show that the virus was limited to the BLA. (**f**) The time spent in the open arms and the time spent in the closed arms in the elevated plus maze test. (**g**) The latency to biting the food and the food intake in the novelty-suppressed feeding test (n = 8/group). (**h**) The time spent in the center arena of the open field box. (**i,j**) The locomotor activity of mice in the open field test. Vertical bars represent the mean ± SEM. The asterisks indicate significant differences from the relevant control. *P < 0.05, **P < 0.01,***P < 0.001, two-way ANOVA test with one factor as repeated measure for 1b, 1c and 1f, Student’s t test for 1g–1j.

To test whether anxiety-like behaviors depend on endogenous ET1 levels in the amygdala or other regions, we first capitalized on previous studies showing that there can be considerable variability in anxiety levels within mouse strains^37, 38^. Mice underwent the EPM test, and ‘high-anxiety’ and ‘low-anxiety’ mice were in the bottom or top 44%, respectively, in time spent in the open arms of the EPM (i.e., the middle 12% of mice were excluded). These two groups differed significantly on the test day: high-anxiety mice spent more time in the closed arms (F_1,8_ = 84.785, P < 0.001, Fig. 1b) and less time in the open arms (F_1,8_ = 38.434, P < 0.001, Fig. 1b).

Real-time quantitative fluorescence PCR analysis revealed that the level of ET1 mRNA was significantly lower in the amygdala (mainly from BLA) in high-anxiety mice than in low-anxiety mice (F_1,8_ = 9.063, P = 0.017, Fig. 1c). However, the levels of ET1 mRNA in the cortex (mainly the piriform cortex) were not significantly different between the two groups (F_1,8_ = 0.614, P = 0.456, Fig. 1c). The effect of interaction was not significant (F_1,8_ = 3.182, P = 0.112, Fig. 1c). These data indicate that there are anxiety-related differences associated with ET1 expression levels in the amygdala and that down-regulation of ET1 signaling might underlie the mechanism through which the anxiogenic process occurs.

### Neutralizing endogenous ET1 in the BLA enhanced anxiety-like behaviors

To further determine whether ET1 modulates anxiety, we examined the effect of manipulating ET1 activity in the BLA on anxiety-like behaviors, which are well-established functions of the amygdala. LV-ET1 shRNA particles were used to knock down endogenous ET1 gene expression, and C57BL/6 mice were randomly allocated into two groups according to intra-BLA cortical treatment (control lentivirus or LV-ET1 shRNA). The control virus was a null lentivirus with no shRNA expression. Figure 1d shows the experimental protocol. Two weeks after virus infusion, mice underwent the following anxiety-like behavioral tests: the open field test, the elevated plus maze test, and the novelty-suppressed feeding test (which entailed food deprivation for the previous 24 h). After each behavioral test, mice were allowed to rest for 3–4 d.

Figure 1e demonstrates that all of the syringes were bilaterally implanted into the BLA. In our previous work, it was confirmed that the spread of the drugs was limited only to the BLA^6^. Figure S2a shows that the ET1 mRNA expression level in the BLA was significantly reduced by LV-ET1 shRNA treatment. However, ET1 mRNA expression in the neighboring regions (piriform cortex or CeA, Figure S2e and i) was not changed by LV-ET1 shRNA treatment. Figure S5 shows that the expression of the control virus was limited to the BLA. Infusing LV-ET1 shRNA into the BLA decreased the time spent in the open arms (F_1,14_ = 80.029, P < 0.001, Fig. 1f) and increased the time spent in the closed arms (F_1,14_ = 26.808, P < 0.001, Fig. 1f). The effect of interaction was significant (F_1,14_ = 16.775, P = 0.001, Fig. 1f). Similar anxiogenic effects of ET1 gene knock-down were also observed in the novelty-suppressed feeding latency test: mice treated with LV-ET1 shRNA showed increased latency to biting the food (F_1,14_ = 11.862, P = 0.004, Fig. 1g) without the food intake amount being affected (F_1,14_ = 0.088, P = 0.771, Fig. 1g). Additionally, mice treated with LV-ET1 shRNA that were subjected to the open field test spent less time in the central area than control mice [t(14) = 2.506, P = 0.025, Fig. 1h]. However, BLA ET1 gene knock-down did not affect the activity of the mice, as indicated by total path length and speed in the open field test [t(14) = −0.212 and −0.875, P = 0.835 and 0.397, respectively; Fig. 1i and j].

Moreover, we infused exogenous ET1 peptide into the BLA and found that ET1 attenuated mouse anxiety-like behaviors (Figure S4). Infusing ET1 into the BLA increased the time spent in the open arms (F_1,46_ = 18.412, P < 0.001, Figure S4a) and decreased the time spent in the closed arms (F_1,46_ = 18.758, P < 0.001, Figure S4a). The effect of interaction is significant (F_1,46_ = 21.205, P < 0.001, Figure S4a). Similar anxiolytic effects of ET1 were also observed in the novelty-suppressed feeding latency test: mice infused with ET1 showed decreased latency to biting the food (F_1,22_ = 11.933, P = 0.002, Figure S4b) without affecting the food intake amount (F_1,22_ = 0.019, P = 0.892, Figure S4b). Additionally, mice treated with ET1 that were subjected to the open field test spent more time in the central area than did control mice [t(22) = −6.159, P < 0.001, Figure S4c]. However, ET1 did not affect the activity of the mice, as indicated by total path length and speed in the open field test [t(22) = 1.197 and 6.698, P = 0.295 and 0.841, respectively; Figure S4d and e]. Taken together, these findings suggest that the ET1 signaling pathway in the BLA regulates mouse anxiety-like behaviors.

### Up-regulating ETBR gene expression in the BLA with ETBR lentiviral activation particles (LV-ETBR) attenuated anxiety-related behaviors

**Figure 2:**
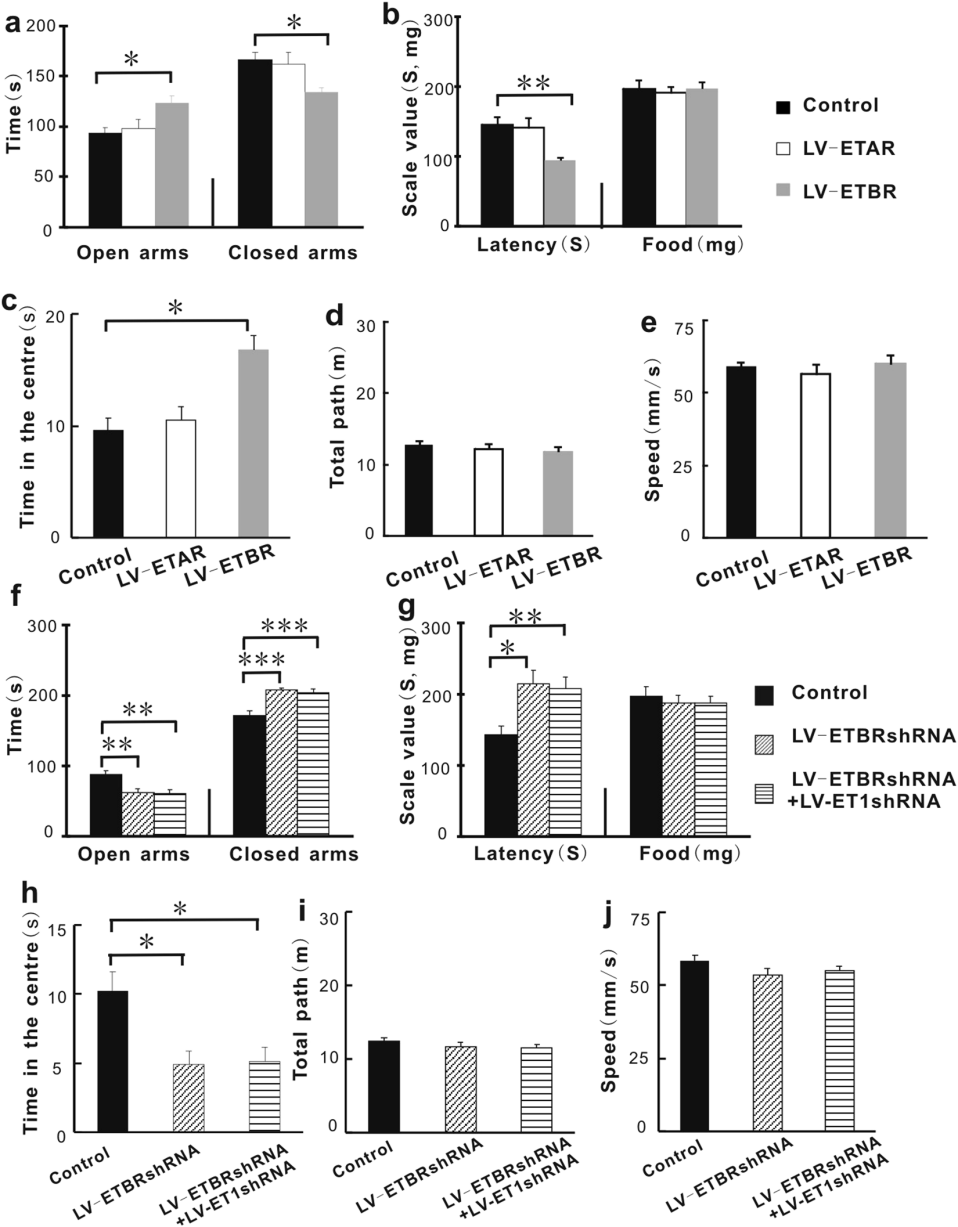
Up-regulating ETBR gene expression in the BLA with ETBR lentiviral activation particles (LV-ETBR) attenuated anxiety-related behaviors. Knocking down ETBR gene expression in the BLA increased anxiety-related behaviors, and down-regulating both ETBR and ET1 had no additional anxiogenic effect. (**a**) The time spent in the open arms and the time spent in the closed arms in the elevated plus maze test. (**b**) The latency time and the food intake of the mice in the novelty-suppressed feeding test. (**c**) The time spent in the center of the open field box. (**d**,**e**) The total path length and the speed in the open field test. (**f**) The time spent in the open arms and the time spent in the closed arms in the elevated plus maze test. (**g**) The latency time and the food intake of the mice in the novelty-suppressed feeding test. (**h**) The time spent in the central area in the open field test. (**i**,**j**) Locomotor activity in the open field test. (n = 16/group). *P < 0.05, **P < 0.01,***P < 0.001, two-way ANOVA test with one factor as repeated measure for 2a and 2f, one way ANOVA post hoc test for 2b–2e and 2g–2i.

To investigate which type of ET1 receptor, ETAR or ETBR, contributes to the regulation of anxiety, we also measured ETAR and ETBR mRNA levels in the same samples used in Figure 1c, and we found that the effects of interactions were not significant in Figure S1b (F1,8 = 0.115, P = 0.743), or in Figure S1c (F1,8 = 2.851, P = 0.030). The cortical mRNA levels of ETAR (F_1,8_ = 0.751, P = 0.411, Figure S1b) and ETBR (F_1,8_ = 1.415, P = 0.268, Figure S1c) were similar between the high-anxiety group and low-anxiety group. The amygdalar mRNA levels of ETBR (F_1,8_ = 15.430, P = 0.004, Figure S1c) but not ETAR (F_1,8_ = 0.436, P = 0.527, Figure S1b) were lower in high-anxiety mice than in low-anxiety mice. To confirm this result, we randomly allocated the mice into three groups based on their BLA treatment: the control lentivirus group, the ETAR lentiviral activation particles (LV-ETAR) group, or the ETBR lentiviral activation particles (LV-ETBR) group. ETAR and ETBR lentiviral activation particles belong to a system for synergistically mediating transcriptional activation designed to specifically and efficiently up-regulate gene expression via lentiviral transduction of cells. The experimental protocol is shown in Fig. 1d. As shown in Figure S2, ETAR mRNA and ETBR mRNA expression levels were significantly increased by LV-ETAR treatment and LV-ETBR treatment, respectively. We found that the performance of the LV-ETAR group did not differ from that of the control group in the elevated plus maze test, in the novelty-suppressed feeding test or in the open field test (Fig. 2). However, the LV-ETBR group showed reduced anxiety-like behaviors: mice spent more time exploring the open arms (F_2,45_ = 4.112; P = 0.033; Fig. 2a) and less time in the closed arms in the elevated plus maze test (F_2,45_ = 4.606; P = 0.021; Fig. 2a), took less time to explore and bite the food in the novelty-suppressed feeding test (F_2,45_ = 6.676; P = 0.005; Fig. 2b), and spent more time in the central region of the open field test box (F_2,45_ = 10.494; P = 0.01; Fig. 2c). The effect of interaction was significant (F_2,45_ = 5.625; P = 0.007; Fig. 2a). Additionally, over-expression of the ETBR gene in the BLA did not decrease the total path length and movement speed (F_2,45_ = 0.674 and 0.453, respectively; P = 0.513 and 0.997, respectively; Fig. 2d and e) or the appetite of the mice (F_2,45_ = 0.0.112; P = 0.999; Fig. 2b). These findings suggest that BLA ET1 regulates anxiety-like behaviors through ETBR but not ETAR.

### Combined down-regulation of ETBR and the ET1 gene has no additional anxiogenic effect compared to knocking down the ETBR gene alone

To further test the above conclusion that the ET1-ETBR contributes regulating anxiety, we used an shRNA lentivirus-mediated gene knock-down technique and compared the effect of treatment with LV-ETBR shRNA (0.25 μl) + LV-ET1 shRNA (0.25 μl) with that of treatment with LV-ETBR shRNA (0.5 μl) alone. The experimental protocol is shown in Fig. 1d. We found that ETBR mRNA expression levels were significantly decreased by LV-ETBR shRNA treatment (Figure S2c) and that ETBR knock-down did not induce compensatory ETAR up-regulation (Figure S2m).

As shown in Fig. 2, BLA ETBR knock-down produced an anxiogenic effect compared with the control lentivirus treatment group: mice spent less time exploring the open arms in the elevated plus maze test (F_2,45_ = 9.961; P = 0.002; Fig. 2f), more time in the closed arms of the elevated plus maze (F_2,45_ = 13.143; P < 0.001; Fig. 2f), more time exploring before biting the food in the novelty-suppressed feeding test (F_2,45_ = 6.373; P = 0.01; Fig. 2g), and less time in the central region of the open field test box (F_2,45_ = 7.868; P = 0.047; Fig. 2h). The effect of interaction was significant (F_2,45_ = 15.210; P < 0.001; Fig. 2f). Additionally, the distance and speed of movement did not differ among the groups (F_2,45_ = 1.1.24 and 1.256, respectively; P = 0.584 and 0.559, respectively; Fig. 2i and j), nor did the appetite of the mice (F_2,45_ = 0.239; P = 0.926; Fig. 2g). Combined down-regulation of ETBR and ET1 gene treatment also produced anxiogenic effects compared with the control: mice spent less time in the open arms of the elevated plus maze test (F_2,45_ = 9.961; P = 0.001; Fig. 2f), more time in the closed arms of the elevated plus maze test (F_2,45_ = 13.143; P < 0.001; Fig. 2f), more time exploring before biting the food in the novelty-suppressed feeding test (F_2,45_ = 6.373; P = 0.008; Fig. 2g), and less time in the center of the open field box (F_2,45_ = 7.868; P = 0.047; Fig. 2h). However, no significant differences were observed between the LV-ETBR shRNA group and the LV-ETBRsh RNA + LV-ET1 shRNA group. These findings confirmed the above conclusion that, in the BLA, ETBR but not ETAR in the BLA may be important for ET1-mediated regulation of anxiety-like behaviors.

### In the BLA, ET1 and its receptors (ETARs and ETBRs) are present mainly in glutamatergic neurons

**Figure 3:**
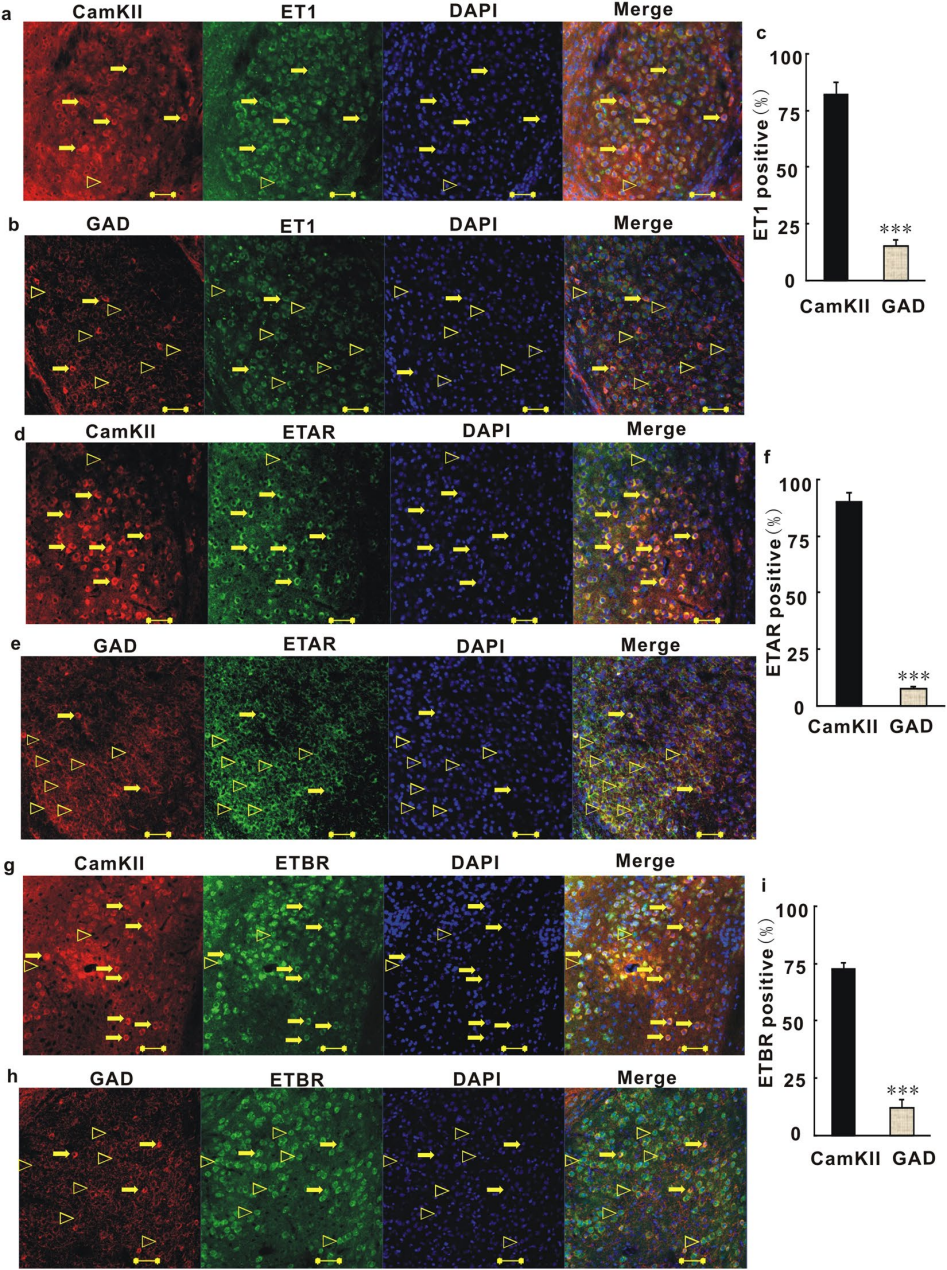
ET1 and its receptors (ETARs and ETBRs) are mainly present at glutamatergic neurons in the BLA. (**a**) Coronal sections of the BLA of C57 mice were stained with anti-CamKII antibody, anti-ET1 antibody and DAPI (blue). Most the right image shows combinations of red (CamKII), green (ET1) and blue (DAPI) channels. Unfilled triangles, ET1-positive neurons without CamKII; arrows, ET1- and CamKII-positive neurons. (**b**) Coronal sections of the BLA were stained with anti-GAD antibody, anti-ET1 antibody and DAPI. Most the right image shows combinations of red (GAD), green (ET1) and blue (DAPI) channels. Unfilled triangles, ET1-positive neurons without GAD; arrows, ET1- and GAD-positive neurons.(**c**) Quantitative analysis of pyramidal neurons and GABAergic neurons that are positive for ET1. (**d**) Coronal sections of the BLA were stained with anti-CamKII antibody, anti-ETAR antibody and DAPI. Most the right image shows combinations of red (CamKII), green (ETAR) and blue (DAPI) channels. Unfilled triangles, ETAR-positive neurons without CamKII; arrows, ETAR- and CamKII-positive neurons. (**e**) Coronal sections of the BLA were stained with anti-GAD antibody, anti-ETAR antibody and DAPI. Most the right image shows combinations of red (GAD), green (ET1) and blue (DAPI) channels. (**f**) Quantitative analysis of pyramidal neurons and GABAergic neurons that were positive for ETAR. Unfilled triangles, ETAR-positive neurons without GAD; arrows, ETAR- and GAD-positive neurons. (**g**) Coronal sections of the BLA were stained with anti-CamKII antibody, anti-ETBR antibody and DAPI. Most the right image shows combinations of red (CamKII), green (ETBR) and blue (DAPI) channels. Unfilled triangles, ETBR-positive neurons without CamKII; arrows, ETBR- and CamKII-positive neurons. (**h**) Coronal sections of the BLA were stained with anti-GAD antibody, anti-ETBR antibody and DAPI. Most the right image shows combinations of red (GAD), green (ETBR) and blue (DAPI) channels. Unfilled triangles, ETBR-positive neurons without GAD; arrows, ETBR- and GAD-positive neurons. (**i**) Quantitative analysis of pyramidal neurons and GABAergic neurons that were positive for ETBR. Scale Bar = 50 µm. Shown are means ± SE; n = 40 for CamKII-positive cells of 10 independent sections; n = 10 for GAD-positive cells of 10 independent sections. The 10 independent sections of each group were randomly selected from 5 mice. ***P < 0.001, Student’s t test for 3c, 3f, 3i.

We next sought to explore which cell type expressing ET1 plays an important role in anxiety. In the CNS, ET1 expression is widely distributed in the cerebral cortex, striatum, hippocampus, amygdala, pituitary gland, and supraoptic and paraventricular nuclei of the hypothalamus, among other regions^21, 39, 40^. However, thus far, there exists no direct or affirmative morphological evidence of cell type-specific ET1 gene expression, especially in the BLA. To address this issue, we began by performing immunohistochemical studies on amygdala sections. To determine the *in vivo* subcellular localization of ET1 in CaMKII-positive neurons, we stained BLA sections with an anti-CaMKII antibody, an anti-ET1 antibody and DAPI. As shown in Fig. 3a, ET1 was mainly detected in CaMKII-positive neurons. Quantitatively, approximately 82.3 ± 5.3% of ET1 in the BLA was expressed in pyramidal neurons (Fig. 3c). Only a small fraction of ET1-positive and DAPI-stained cells were not CaMKII-positive. Next, we stained coronal sections of the BLA with anti-GAD antibody, anti-ET1 antibody and DAPI. As shown in Fig. 3b, a smaller fraction of ET1 was detected in GAD-positive neurons [t(18) = 16.752, P < 0.001, Fig. 3c]. Quantitatively, approximately 15.2 ± 2.8% of ET1 in the BLA was expressed in interneurons (Fig. 3c). In conclusion, these results indicate that most of the ET1 in the BLA is present in neurons and that ET1 is mainly expressed in pyramidal neurons.

Because ET1 acts on two different subtypes of receptors, we next checked the distribution of the ET1 receptor subtypes in the BLA. To explore the ETAR distribution, coronal sections of the BLA were stained with anti-CaMKII antibody (or anti-GAD antibody), anti-ETAR antibody and DAPI. As shown in Fig. 3d, ETARs were mainly detected in CaMKII-positive neurons. Quantitative analysis showed that 90.1 ± 4.3% of ETARs were expressed in BLA pyramidal neurons (Fig. 3f). A smaller amount of ETAR was expressed in BLA GABAergic interneurons [7.63 ± 1.26%, t(18) = 18.516, P < 0.001, Fig. 3f]. Thereafter, ETBR distribution was investigated by staining the BLA sections with anti-CaMKII antibody (or anti-GAD antibody), anti-ETBR antibody and DAPI. As shown in Fig. 3g, ETBRs were mainly detected in CaMKII-positive neurons. Quantitative analysis showed that 72.5 ± 3% of ETBRs were expressed in BLA pyramidal neurons (Fig. 3i). Only a smaller fraction of ETBR was expressed in BLA interneurons [12.2 ± 3.8%, t(18) = 10.22, P < 0.001, Fig. 3h and i]. Taken together, these results indicate that most of the ETARs and ETBRs are distributed in BLA neurons, and that they are mainly expressed in pyramidal neurons, with small fractions of these receptors distributed in BLA interneurons.

### The firing frequency and threshold current of action potential generation are regulated by ET1 and an ETBR antagonist

**Figure 4:**
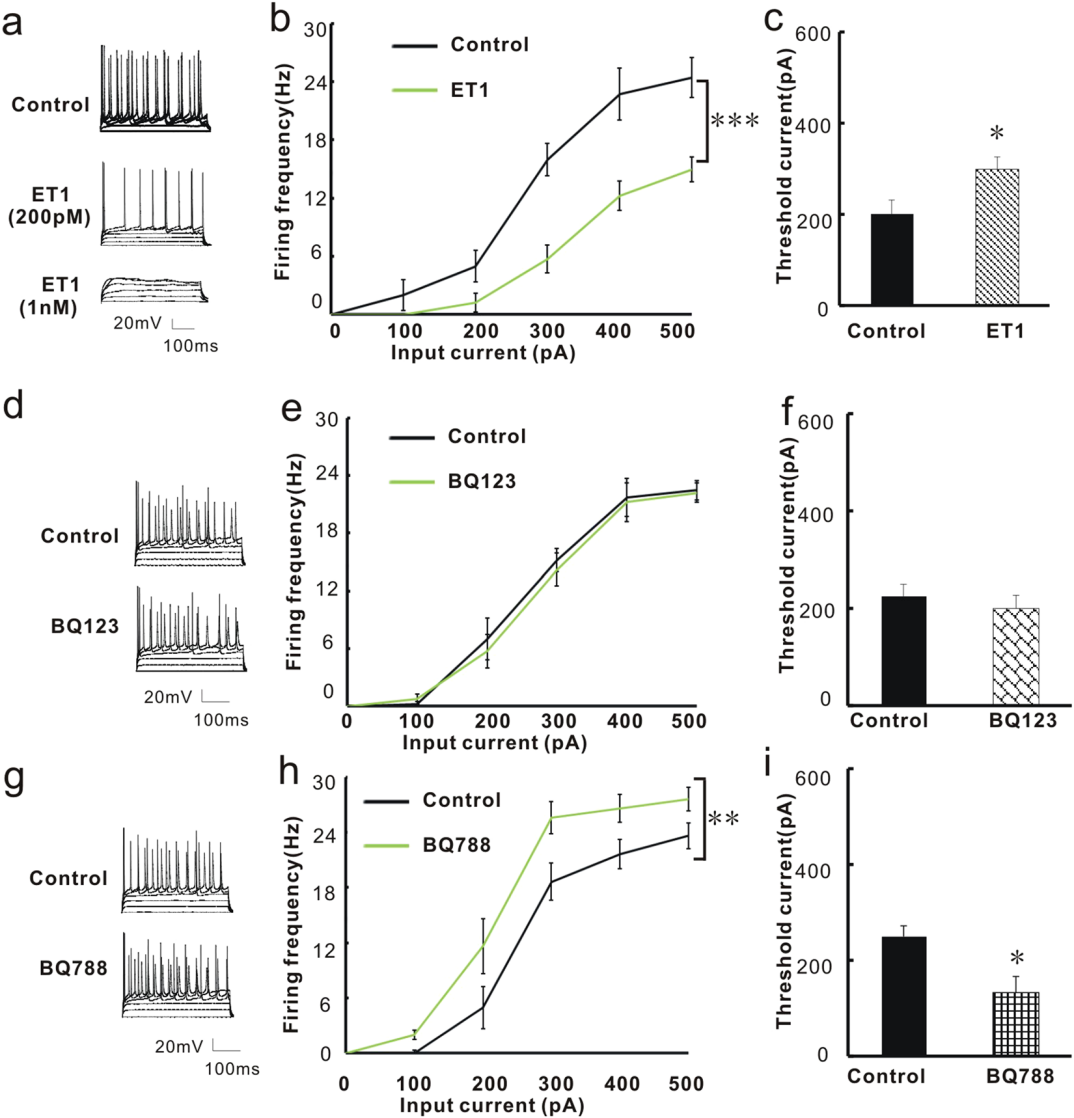
The firing frequency and threshold current of action potential generation are regulated by ET1 and ETBR antagonists. (**a**) Voltage responses of a representative pyramidal neuron in the BLA to current injections of, from bottom to top, 0, 100, 200, 300, 400, and 500 pA. Different concentrations of ET1 solution were applied. (**b**) Summary plot of the firing frequency before and after application of 200 pM ET1 (n = 8/group). (**c**) Summary histogram of the threshold current for action potential before and after application of 200 pM ET1 (n = 8/group). (**d**) Voltage response of a representative pyramidal neuron in the BLA to current injections of, from bottom to top, 0 to 500 pA, before and after BQ123 treatment. (**e**) Summary plot of the firing frequency before and after application of 1 nm BQ123 (n = 8/group). (**f**) Summary histogram of the threshold current for action potential before and after application of 1 nm BQ123 (n = 8/group). (**g**) Voltage response of a representative pyramidal neuron in the BLA to current injections of, from bottom to top, 0 to 500 pA, before and after BQ788 treatment. (**h**) Summary plot of the firing frequency before and after application of 2 nM BQ788 (n = 6/group). (**i**) Summary histogram of the threshold current before and after application of 2 nM BQ788 (n = 6/group). *P < 0.05, two-way ANOVA test with one factor as repeated measure for 4b, 4e and 4 h and Student’s t test for 4c, 4f and 4i.

The finding that ET1 and its receptors are widely expressed in BLA pyramidal neurons suggests that pyramidal neurons are a major cellular target of ET1 signaling in the adult brain. To investigate whether ET1 directly regulates the function of BLA pyramidal neurons, we first performed whole-cell current-clamp recordings in acute BLA slices to identify pyramidal neurons by their pyramid-shaped cell bodies with a single apical dendrite and multiple basal dendrites. The effect of ET1 on the action potential was then investigated. We directly measured the firing frequency and threshold current for action potential generation by probing with currents from 0 to 500 pA in 100-pA steps. Figure 4a,d and g show representative traces. When the injected current was increased, all pyramidal neurons showed high-frequency discharges. ET1 treatment (200 pM) suppressed the excitability of pyramidal neurons, as shown by the decreased firing frequencies (F_1,70_ = 24.863, P < 0.001, Fig. 4b) and the increased threshold current [t(14) = −2.366, P = 0.033, Fig. 4c]. No significant interaction was observed in Fig. 4b (F1,70 = 2.587, P = 0.096). Action potentials were completely blocked with the application of 1 nM ET1 (Fig. 4a). However, neither the frequency (F_1,70_ = 0.122, P = 0.732, Fig. 4e) nor the threshold current [t(14) = 0.683, P = 0.506, Fig. 4f] was changed by the application of an ETAR antagonist (BQ123, 1 nM). No significant interaction was observed in Fig. 4e (F1,70 = 0.142, P = 0.963). We then evaluated the effect of an ETBR antagonist (BQ788, 2 nM) on the excitability of BLA pyramidal neurons, and we found the effect was opposite to that described for the application of ET1 (Fig. 4g–i). BQ788 significantly increased the excitability of pyramidal neurons, as shown by the increased firing frequencies (F_1,50_ = 19.985, P = 0.001; Fig. 4h). No significant interaction was observed in Fig. 4h (F1,50 = 2.1, P = 0.184). BQ788 also decreased the threshold current for action potential generation [t(10) = 2.907, P = 0.016; Fig. 4i]. These results indicated that the ET1/ETBR signaling pathway influences the excitability of pyramidal neurons by regulating the action potential threshold.

### ET1 regulates the excitability of BLA pyramidal neurons through ETBRs

**Figure 5:**
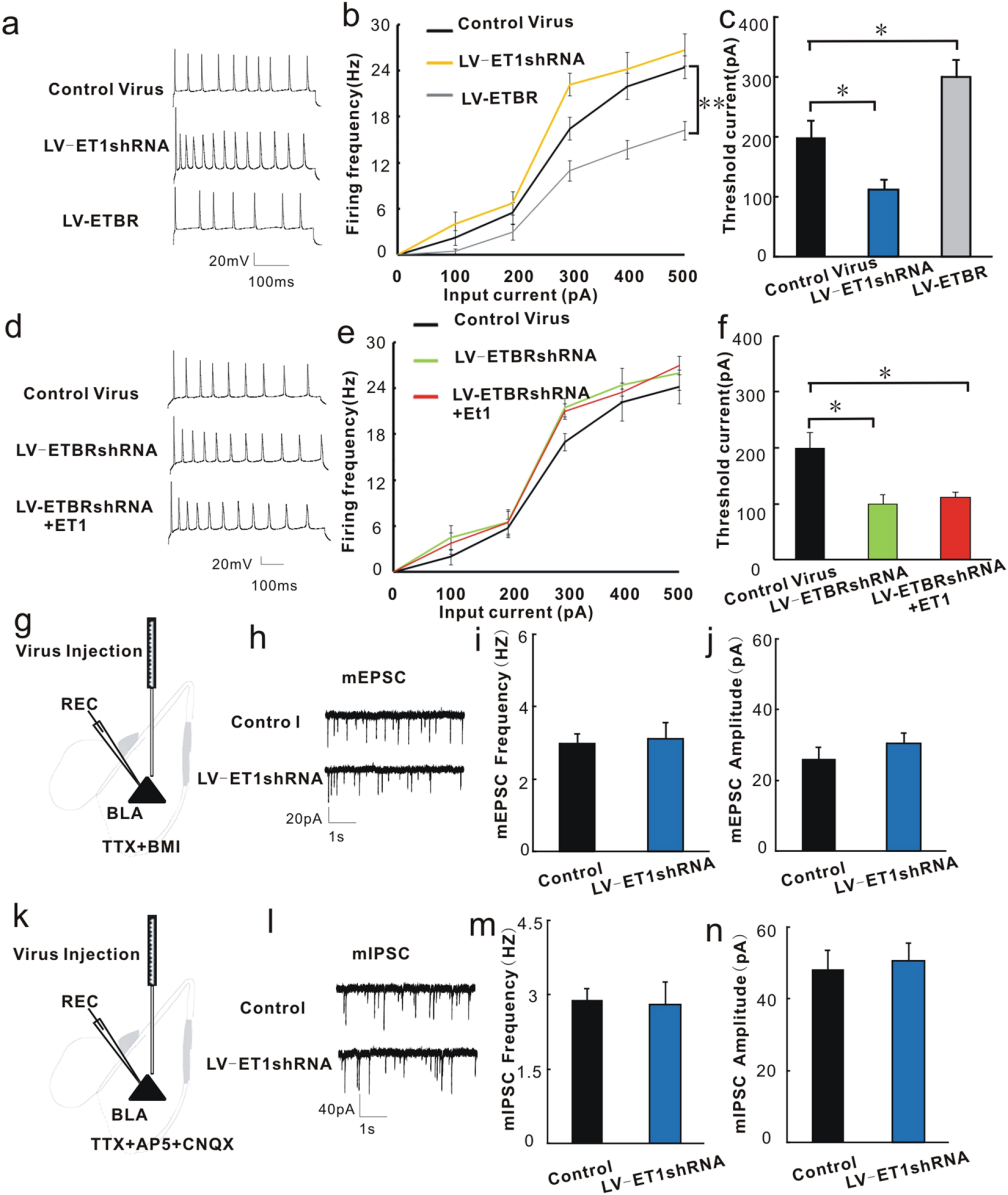
ET1 directly regulates the excitability of BLA pyramidal neurons through ETBRs. Viruses were injected into the BLA two weeks before the patch-clamp recordings. (**a**) Representative action potentials of pyramidal neurons in the BLA from mice treated with the control lentivirus, LV-ET1 shRNA and LV-ETBR. (**b**) Summary histogram of the firing frequency of action potentials. (**c**) Summary histogram of the threshold current for action potential generation. (**d**) Representative action potentials of pyramidal neurons in the BLA from slices treated with the control lentivirus, LV-ETBR shRNA and LV-ETBR shRNA + ET1 (probing with 300 pA current). (**e**) Summary histogram of the firing frequency of action potentials. (**f**) Summary histogram of the threshold current for action potential generation (n = 8/group). (**g**) Schematic illustration of the injection sites for virus in the BLA and recording sites for the mEPSCs of BLA pyramidal neurons. (**h**) Representative mEPSC traces of BLA pyramidal neurons treated with control lentivirus or LV-ET1 shRNA. (**i**) Summary histogram of the effect of LV-ET1 shRNA on the mEPSC frequencies of BLA pyramidal neurons. (**j**) Summary histogram of the effect of LV-ET1 shRNA on mEPSC amplitudes of BLA pyramidal neurons (n = 6/group). (**k**) Schematic illustration of the injection sites for LV-ET1 shRNA in the BLA and the recording sites for the mIPSCs of BLA pyramidal neurons. (**l**) Representative mIPSC traces of BLA pyramidal neurons treated with control lentivirus or LV-ET1 shRNA. (**m**) Summary histogram of the effect of LV-ET1 shRNA on mIPSC frequencies of BLA pyramidal neurons. (**n**) Summary histogram of the effect of LV-ET1 shRNA on mIPSC amplitudes of BLA pyramidal neurons (n = 6/group). Triangles (▴) represent pyramidal neurons. *P < 0.05, two-way ANOVA test with one factor as repeated measure for 5b and 5e, one-way ANOVA with post hoc test for 5c and 5f, student’s t test for 5m-5i and 5i–5j.

To further test whether mice with modulated ET1 and ETBR gene expression also show changed excitability of BLA pyramidal neurons, we performed whole-cell current-clamp recordings in acute BLA slices 2 weeks after bilateral injection of viruses into the BLA,. We first measured the threshold current for action potential generation and the firing frequency in BLA pyramidal neurons. Figure 5a shows representative traces for the action potentials of the control lentivirus group, the LV-ET1 shRNA group, and the LV-ETBR group. The LV-ET1 shRNA treatment increased the excitability of pyramidal neurons compared with control virus treatment, as shown by the increased firing frequencies (F_1,70_ = 6.592; P = 0.012, Fig. 5b) and the decreased threshold current (F_2,21_ = 16.476; P = 0.046; Fig. 5c). Over-expression of ETBR decreased the excitability of pyramidal neurons compared with the control group, as shown by the decreased firing frequencies (F_1,70_ = 43.725; P < 0.001; Fig. 5b) and increased the threshold current (F_2,21_ = 16.476; P = 0.039; Fig. 5c). No significant interaction was observed in Fig. 5b (F1,70 = 1.848, P = 0.098). This result is consistent with the pharmacological result shown in Fig. 4 and confirms that ET1-ETBR directly regulates the excitability of BLA pyramidal neurons via a genetic mechanism.

To directly investigate whether ET1 regulates the excitability of BLA pyramidal neurons through ETBRs, we first measured the effect of knocking down the ETBR gene via LV-ETBR shRNA transfection on the excitability of BLA pyramidal neurons, and we then evaluated whether adding ET1 to the incubation solution of LV-ETBR shRNA-transfected BLA slices would produce the opposite effect. Fig. 5d shows representative traces of action potentials of the control lentivirus group, the LV-ETBR shRNA group, and the LV-ETBR shRNA + ET1 group. The LV-ETBR shRNA treatment increased the excitability of pyramidal neurons, as shown by the increased firing frequencies (F_1,70_ = 4.489; P = 0.038; Fig. 5e) and decreased the threshold current (F_2,21_ = 8.489; P = 0.023; Fig. 5f). Perfusing the ETBR knock-down slices with ET1 did not produce an opposite effect compared with LV-ETBR shRNA group. Perfusing the ETBR knock-down slices with ET1 could also increase firing frequencies (F_1,70_ = 4.489; P = 0.038; Fig. 5e) and decrease threshold current (F_2,21_ = 8.489; P = 0.037; Fig. 5f). No significant interaction was observed in Fig. 5e (F_1,70_ = 0.843, P = 0.571). This result is consistent with the above results and further confirms the conclusion that ET1 can regulate the excitability of BLA pyramidal neurons through ETBRs.

To further test whether the changed action potentials of BLA pyramidal neurons mediated by ET1 are due to excitatory synaptic neurotransmission, we injected the control virus and LV-ET1 shRNA into the BLA and measured the miniature excitatory postsynaptic currents (mEPSCs) of BLA pyramidal neurons. mEPSCs represent the excitatory synaptic neurotransmission. Figure 5g shows schematic illustration of the injection sites of the virus into the BLA and the recording sites for BLA pyramidal neurons. The incubation solution contained TTX and BMI to block the action potentials and inhibitory neurotransmission of BLA cells. Figure 5h shows representative mEPSC traces of BLA pyramidal neurons treated with the control virus or LV-ET1 shRNA treatment. Neither the frequencies [t(10) = −0.251, P = 0.807, Fig. 5i] nor the amplitudes [t(10) = −0.923, P = 0.38; Fig. 5j] of mEPSCs were affected by ET1 knock-down. These results suggested that ET1 regulates the action potentials of BLA pyramidal neurons, which is not due to excitatory synaptic neurotransmission.

The above immunohistochemical results show that there are small amounts of ET1 distributed in the interneurons. To test whether ET1 regulates inhibitory neurotransmission, we injected the LV-ET1 shRNA into the BLA and evaluated the miniature inhibitory postsynaptic currents (mIPSCs) of BLA pyramidal neurons. mIPSCs represent inhibitory synaptic neurotransmission. Figure 5k shows a schematic illustration of the injection sites of the virus into the BLA and the recording sites for BLA pyramidal neurons. The incubation solution contained AP5, CNQX and TTX to block the excitatory neurotransmission and action potentials of BLA cells. Figure 5l shows representative mIPSC traces of BLA pyramidal neurons treated with the control lentivirus or LV-ET1 shRNA. LV-ET1 shRNA treatment also induced no effects on mIPSC frequencies or amplitudes [t(10) = 0.201 and −0.341 P = 0.845 and 0.741, Fig. 5m and n, respectively). These results suggest that BLA GABAergic neurotransmission is not regulated by ET1. Altogether, these observations demonstrated that the mechanism underlying the anxiolytic effect of ET1 in the BLA involves a direct decrease in the excitability of BLA pyramidal neurons but does not change excitatory or inhibitory synaptic neurotransmission.

## Discussion

Patients with generalized anxiety disorder may have abnormal activity arising from the amygdala^6, 12^. However, in contrast to the cortex and hippocampus, the pathological and molecular mechanisms of anxiety in the amygdala are not well understood. Although evidence has shown that ET1 is expressed in the amygdala^39^, thus far, no studies have investigated the role of amygdalar ET1 in regulating anxiety. The cell type-specific distribution of ET1 in different brain regions (especially in the amygdala, which is a key brain area for anxiety) and the direct neuronal excitability mechanism mediated by ET1 in the regulation of anxiety also remain unclear.

To the best of our knowledge, this is the first report showing that the ET1-ETBR signaling in the amygdala is important for modulating anxiety-like behaviors by regulating the excitability of BLA pyramidal neurons. The mRNA expression levels of ET1 and ETBR were down-regulated specifically in the amygdala of high-anxiety mice. Most of the ET1 and its receptors were expressed in BLA pyramidal neurons. Down-regulating ET1 expression simultaneously enhanced anxiety-like behaviors and increased the excitability of BLA pyramidal neurons. Over-expression of ETBRs produced anxiolytic effects and decreased the firing rate of BLA pyramidal neurons. Knocking down the ETBR gene counteracted the ET1-induced decrease in action potentials. By contrast, manipulating ETARs produced no effects on these behaviors or on neuronal excitability, suggesting that BLA ET1 acts through ETBRs, but not through ETARs, to regulate neuronal excitability and anxiety-like behaviors. We also found that ET1 did not regulate excitatory synaptic neurotransmission or inhibitory neurotransmission in the BLA. We uncovered a novel function of BLA ET1-ETBR signaling in the regulation of anxiety-like behaviors, a finding that may provide researchers with a new perspective with respect to treating anxiety disorders.

Numerous studies have used animal models to examine candidate genes for their involvement in anxiety-related behavior^41^. In this study, we first used the elevated plus maze test to discriminate innate extremes in anxiety-related behaviors, and then we examined the ET1 gene for involvement in anxiety-related behaviors. In many ways, acute mouse selection models using the elevated plus maze test appear to be superior to, for instance, targeted gene knockouts, because they select mice based on the entire spectrum of neurobiological mechanisms and pathways rather than on one specific altered gene and gene product. Importantly, these acute selection animal models maintain the integration of underlying signaling systems intact. The genetic variability of ET1 may at least partly explain inter-species differences and inter-individual variation in anxiety-related behaviors, making the ET1 system not only an important substrate for the evolution of anxiety behavior but also a promising target for therapeutic interventions.

Unlike our previous work, in which infusion of exogenous ET1 into the infralimbic cortex increased anxiety due to its direct effect of increasing excitatory synaptic neurotransmission through ETARs^21^, here, we demonstrated a new role for ET1, specifically in the BLA, in the regulation of anxiety behavior. We found that BLA ET1 decreased the excitability of BLA pyramidal neurons and had an anxiolytic effect through ETBRs but not through ETARs. We also found that unlike the promoting effect of infralimbic ET1 on synaptic neurotransmission, BLA ET1 did not regulate synaptic neurotransmission. We thus suspect that there is a balance between the infralimbic ET1-ETAR and the BLA ET1-ETBR pathways in controlling anxiety through mechanisms mediated by the different receptors. The pleiotropic effects of ET1 are based on its different receptor-mediated modes of interneuronal communication in distinct brain areas. ET1 signals through two G protein-coupled receptors, ETAR and ETBR, and can lead to the activation of a variety of signaling cascades, such as the nuclear factor-kappa-binding (NF-κB), β-catenin, phosphoinositide 3-kinase, and mitogen-activated protein kinase signaling cascades^42^. According to previous studies, an ETBR agonist elicited calcium mobilization with β-catenin and NF-κB signaling, and ETAR activation led to transforming growth factor-β (TGF-β) production^42^. Evidence from pharmacological and genetic studies of the NF-κB complex in relation to anxiety-related behaviors and stress suggests that the NF-κB complex is indeed involved in emotional behavior and stress responses^43, 44^. The implications of TGF-β–related findings for the molecular understanding and potential treatment of neuropsychiatric diseases, such as anxiety, depression and other neurological disorders, have also been discussed^45^. Differences in the downstream signaling pathways of ETAR and ETBR may account for the differences in the effects of infralimbic ET1 and BLA ET1 on anxiety. The anxiolytic effect of the BLA ET1-ETBR signaling pathway, through activating the TGF-β, may counter the anxiogenic effect of the infralimbic ET1-ETAR signaling pathway, through activating the NF-κB complex, in normal mice, and the imbalance of these pathways would lead to anxiety disorders.

The involvement of ET1 in neuroendocrine stress regulation (hypothalamic-pituitary-adrenal, HPA axis), and its interaction with other neuropeptides provide the potential for feed-forward effects, presumably balancing and adjusting biologically adequate behavior in opposite manners. Among these other neuropeptides, corticotropin-releasing hormone (CRH) and vasopressin are of particular interest. The HPA axis is responsible for the coordination of the neuroendocrine, autonomic, and behavioral responses to stress. Systemically administered ET1 stimulated the HPA axis, leading to CRH release^46^. Further studies have shown that CRH and ET1 regulate each other^47^. Vasopressin has also been demonstrated to induce ET1 release^48^. The anxiogenic effects of CRH and vasopressin may be initially beneficial to the individual, serving to adjust behavior and physiology to increase short-term survival, possibly at the potential expense of increasing susceptibility to disease over the long term. Thus, the anxiolytic role of amygdala ET1 is the body’s self-protection mechanism that likely counteracts the long-term anxiogenic effects of CRH and vasopressin, thereby leading to far-reaching functional stability.

ET1 is a vasopressive protein that causes local vasoconstriction around injection sites, resulting in ischemia-induced tissue damage in affected areas^49, 50^. We therefore tried to identify ET1 concentrations below the threshold for vascular effects. According to our previous study^21^, we applied ET1 (1 nM to 1 μM) into the bath and assessed changes in both the threshold currents and the frequencies of action potentials. However, at these concentrations, we observed significant vasoconstriction of large blood vessels in the BLA, which impaired the stability of the patch-clamp recordings. At ET1 concentrations of ≦ 200 pM, the slices were relatively stable, enabling us to record for long periods of time (>30 min) without unacceptable changes in whole-cell access resistances. The present study provides compelling evidence that ET1, at concentrations below the threshold for vascular effects, can modulate the excitability of BLA pyramidal neurons. Moreover, we also found that at ET1 concentrations of ≧1 nM, the action potentials of BLA pyramidal neurons were totally blocked, but the slices were also unstable, which means that an overdose of ET1 could cause both neuronal death and vasoconstriction. These results suggest that ET1 has a two-phase effect: at low doses, it has an anxiolytic effect, whereas at high doses, it has a negative effect that could cause neuronal death and stroke. Additional investigations should be conducted to extend the current results.

As a potent vasoconstrictor, ET1 has been extensively studied in stroke. Here, we also conducted deeper investigations into ET1 as a potential shared genetic risk factor for anxiety disorder. The etiopathological significance and treatment implications of ET1 for anxiety and stroke are beneficial and far-reaching. ET1 antagonists are now likely to emerge as important therapeutic strategies for the treatment of several cardiovascular and other diseases. Indeed, the effects of these antagonists on the CNS have not been fully investigated. Our results emphasize the importance of better understanding ET1-mediated anxiolytic effects and provide some useful information for further clinical investigations on the function of ET1 in the CNS, which may help physicians to take precautions against the side effects of ET1-specific targeted drugs.

## Electronic supplementary material

Supplementary data
