# Supplementary material for: Amygdalar Endothelin-1 Regulates Pyramidal Neuron Excitability and Affects Anxiety
Source: Sci Rep. 2017 May 24;7:2316. doi: 10.1038/s41598-017-02583-6 (PMC5443782; doi:10.1038/s41598-017-02583-6)
Supplement: Supplementary file 1 — Supplementary data [file 41598_2017_2583_MOESM1_ESM.doc]

**Amygdalar Endothelin-1 Regulates Pyramidal Neuron Excitability and Affects Anxiety**

Running Title: Amygdalar Endothelin1-mediated Regulation of Anxiety

Ming Chen^1,3^, Huan-huan Yan^4^, Shu Shu^4^, Lei Pei^4^, Long-kai Zang^2^, Yu Fu^2^, Ze-fen Wang^5^, Qi Wan^5^ and Lin-lin Bi*^,1,2^

^1^Wuhan University Center for Pathology and Molecular Diagnostics, Zhongnan Hospital of Wuhan University, Wuhan 430071, China

^2^Department of Pathology, Wuhan University School of Basic Medical Sciences, Wuhan 430071, China

^3^Department of Cardiology, Zhongnan Hospital of Wuhan University, Wuhan 430071, China

^4^Department of Pathophysiology and Key Laboratory of Neurological Diseases of Ministry of Education, Tongji Medical College, Huazhong University of Science and Technology,Wuhan430030, China

^5^Department of Physiology, Wuhan University School of Basic Medical Sciences, Wuhan 430071, China

^*^To whom correspondence may be addressed. E-mail: linlinbi2016@whu.edu.cn

**Intracerebral infusions**

As previously reported^6^, mice were anesthetized with an intraperitoneal injection of chloral hydrate (400 mg/kg) and placed into a stereotaxic instrument (Stoelting, USA). The mouse scalp was removed, and small burr holes were drilled into the skull (1 mm diameter) with a drill. Stainless-steel guide cannulas (Plastics One; C315G/SPC; length, 6 mm) were lowered into the random and unilateral BLA using the following coordinates, anterior, -1.5mm; lateral, ±3.2mm; and ventral, -4.7 mm, according to The Mouse Brain in Stereotaxic Coordinates. The guide cannulas were fixed in place with glass ionomer cements. Dummy cannulas (Plastics One, INC; C315DC/SPC, length matched to the guide cannulas) were placed inside the guide cannulas to prevent occlusion. Seven days after surgery, drug infusions and the behavioral tests were performed.

To perform drug infusions, the dummy cannulas were removed quickly from the guide cannulas and replaced by infusion cannulas (Plastics One, INC; C315I/SPC, lengths matched to the guide cannulas). The infusion cannulas were connected, via polyethylene tubing (Plastics One, INC; C313C), to 10 μl microsyringes (Hamilton, Reno, NV) mounted on a microinfusion pump (RWD200, China). The injection amount was 0.5 μl/side, and the rate was 0.25 μl/min. After that, the infusion cannulas were kept in place for another two minutes to allow diffusion of the drug before being replaced with the dummy cannulas. One hour after the drug infusion, the behavioral tests were performed.


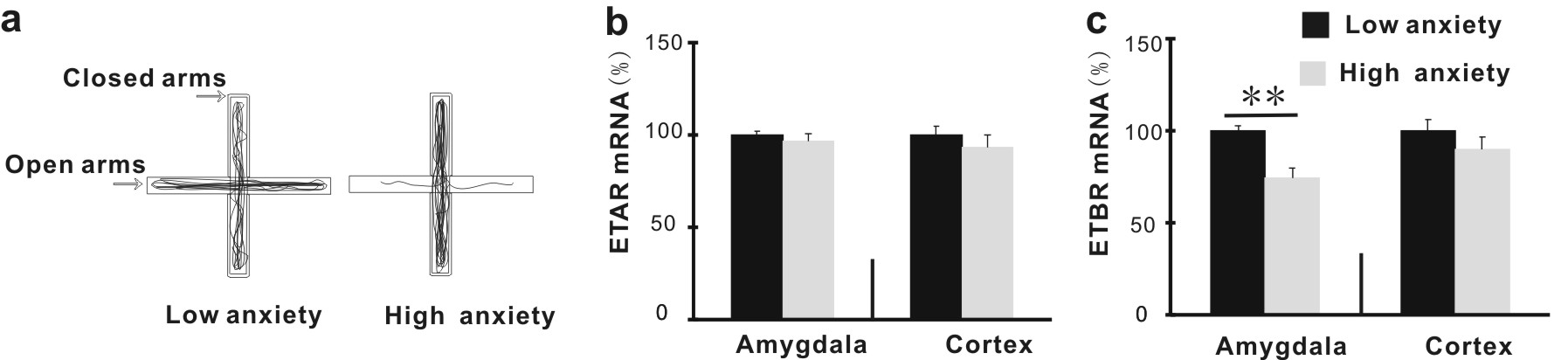


Figure S1 High-anxiety mice show lower amygdalar mRNA levels of ETBR, but not of ETAR, compared with low-anxiety mice. (a) Schematic illustration of the elevated plus maze test. The left trace represents the paths of low-anxiety mice, and the right trace represents the paths of high anxiety mice. (b) Real-time quantitative fluorescence PCR analysis showing ETAR mRNA expression. (c) Real-time quantitative fluorescence PCR analysis showing ETBR mRNA expression. n=5/group, **P<0.01, two-way ANOVA test with one factor as repeated measure for b and c.


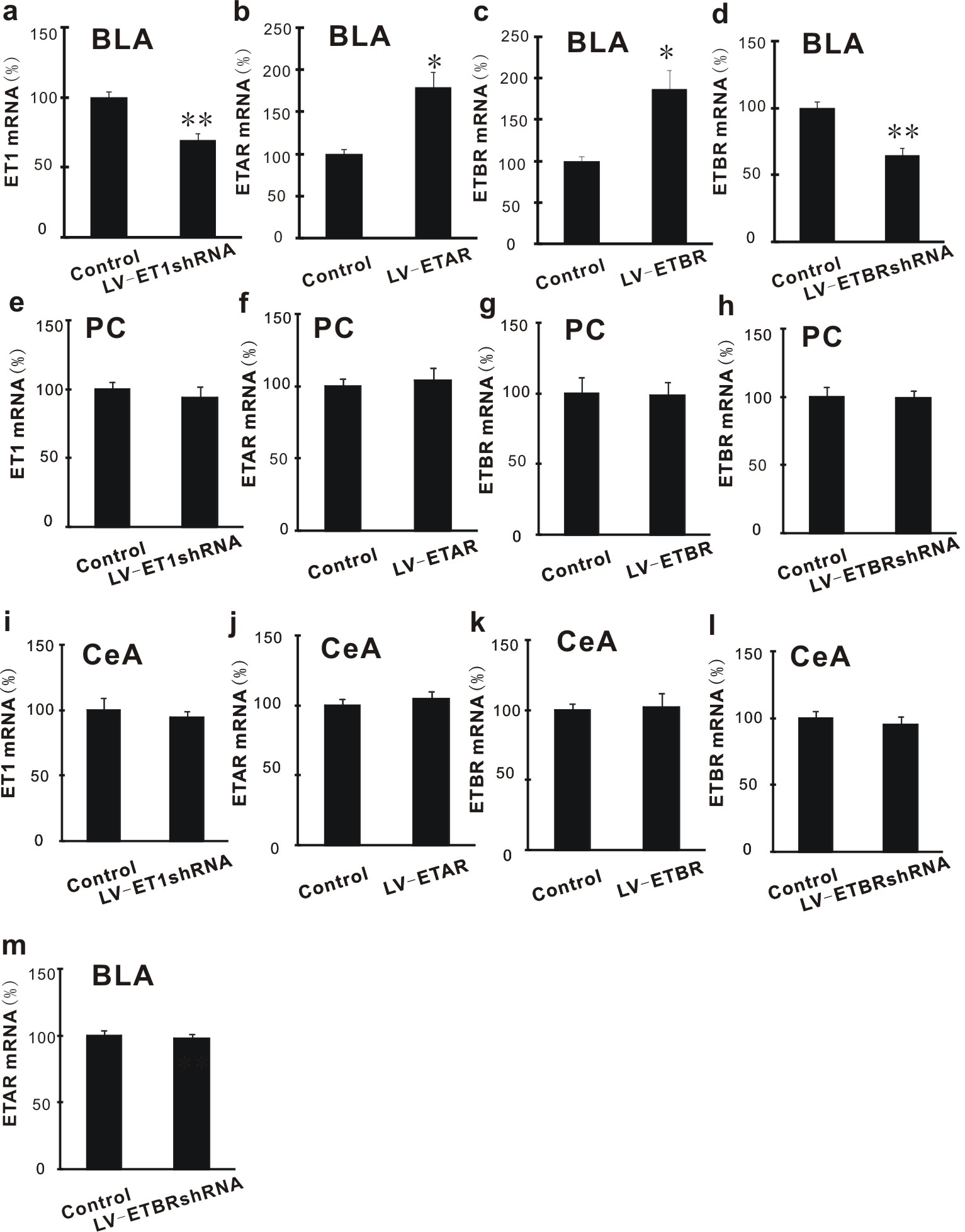


Figure S2 (a-d) RT-PCR analysis for mRNA from the BLA tissue. (e-h) RT-PCR analysis for mRNA from the piriform cortex (PC) tissue. (a-d, m) RT-PCR analysis for mRNA from the central amygdala (CeA) tissue. The results of real-time RT-PCR showing the changes in ET1 mRNA levels two weeks after infusion of LV-ET1shRNA into the BLA (a, e, i), in ETAR mRNA levels two weeks after infusion of LV-ETAR into the BLA (b, f, j), ETBR mRNA levels two weeks after infusion of LV-ETBR into the BLA (c, g, k), ETBR mRNA levels two weeks after infusion of LV-ETBRshRNA into the BLA (d, h, l), ETAR mRNA levels two weeks after infusion of LV-ETBRshRNA into the BLA (b, f, j), and ETAR mRNA levels two weeks after infusion of LV-ETBRshRNA into the BLA (d, h, l),. (*P<0.05, **P<0.01, student’s t test)


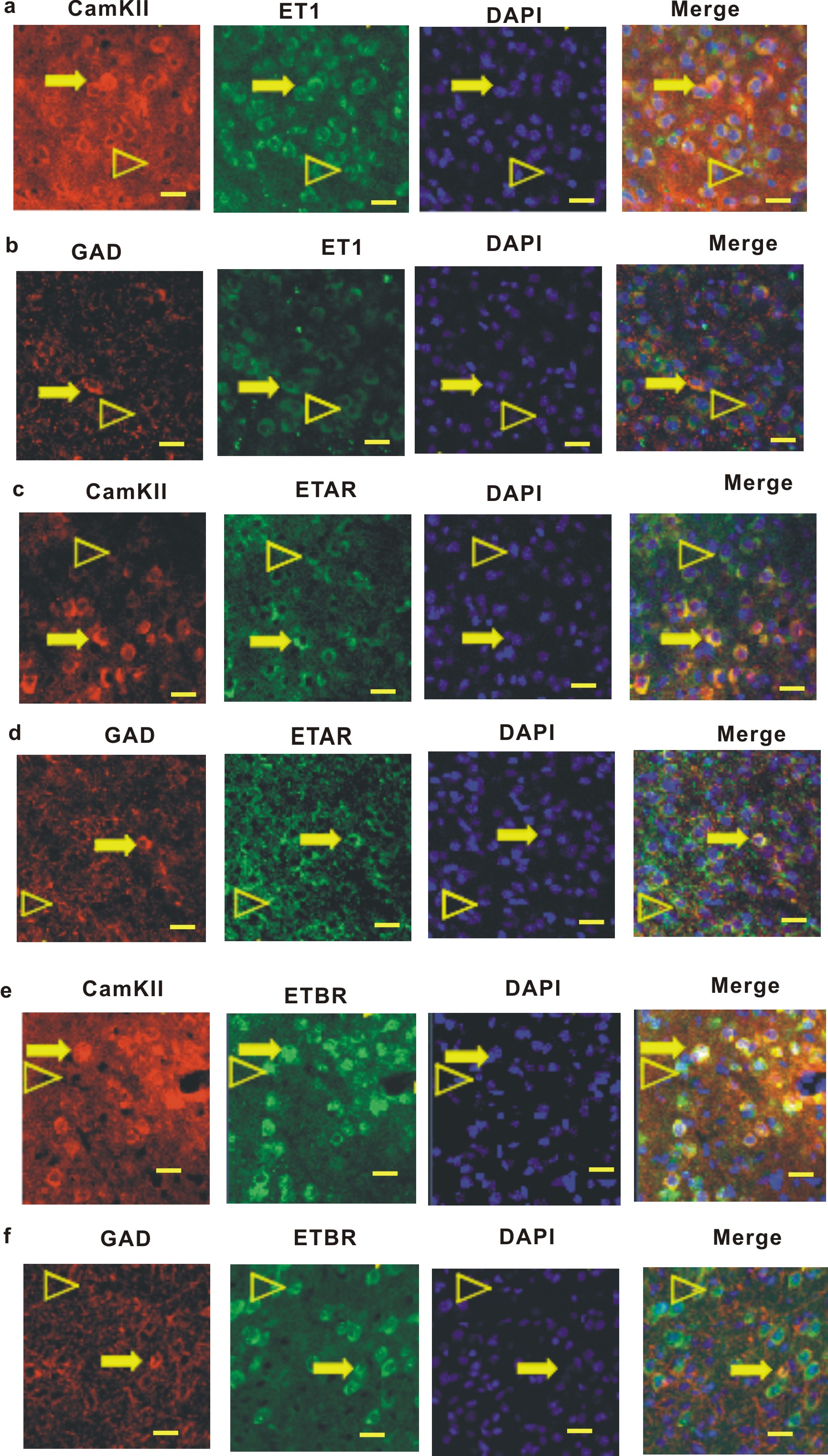


Figure S3 ET1 and its receptors (ETARs and ETBRs) are mainly present at glutamatergic neurons in the BLA. (a) Coronal sections of the BLA of C57 mice were stained with anti-CamKII antibody, anti-ET1 antibody and DAPI (blue). Immunoactivity of CamKII was visualized by Alexa 594-conjugated secondary antibody (Red). Immunoactivity of ET1 was visualized by Alexa 488-conjugated secondary antibody (Green). Most the right image shows combinations of red (CamKII), green (ET1) and blue (DAPI) channels. Unfilled triangles, ET1-positive neurons without CamKII; arrows, ET1- and CamKII-positive neurons. (b) Coronal sections of the BLA were stained with anti-GAD antibody, anti-ET1 antibody and DAPI. Immunoactivity of GAD was visualized by Alexa 594-conjugated secondary antibody. Most the right image shows combinations of red (GAD), green (ET1) and blue (DAPI) channels. Unfilled triangles, ET1-positive neurons without GAD; arrows, ET1- and GAD-positive neurons. (c) Coronal sections of the BLA were stained with anti-CamKII antibody, anti-ETAR antibody and DAPI. Immunoactivity of ETAR was visualized by Alexa 488-conjugated secondary antibody. Most the right image shows combinations of red (CamKII), green (ETAR) and blue (DAPI) channels. Unfilled triangles, ETAR-positive neurons without CamKII; arrows, ETAR- and CamKII-positive neurons. (d) Coronal sections of the BLA were stained with anti-GAD antibody, anti-ETAR antibody and DAPI. Most the right image shows combinations of red (GAD), green (ET1) and blue (DAPI) channels. (e) Coronal sections of the BLA were stained with anti-CamKII antibody, anti-ETBR antibody and DAPI. Immunoactivity of ETBR was visualized by Alexa 488-conjugated secondary antibody. Most the right image shows combinations of red (CamKII), green (ETBR) and blue (DAPI) channels. Unfilled triangles, ETBR-positive neurons without CamKII; arrows, ETBR- and CamKII-positive neurons. (f) Coronal sections of the BLA were stained with anti-GAD antibody, anti-ETBR antibody and DAPI. Most the right image shows combinations of red (GAD), green (ETBR) and blue (DAPI) channels. Unfilled triangles, ETBR-positive neurons without GAD; arrows, ETBR- and GAD-positive neurons. Means±SEM are shown; more than 1000 clusters of five independent sections were scored for each group. Scale Bar=25 µm.


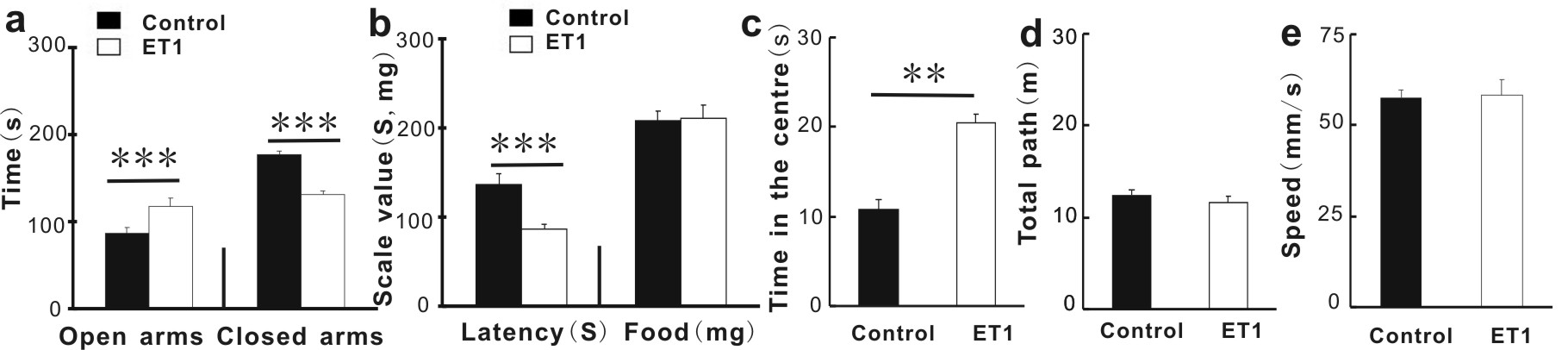


Figure S4 Infusing ET1 peptide into the BLA attenuated anxiety-like behaviors. (a) The time spent in the open arms and the time spent in the closed arms in the elevated plus maze test. (b) The latency time to biting the food and the food intake in the novelty-suppressed feeding test. (c) The time spent in the center arena of the open field box. (d, e) The locomotor activity of mice for the open field test. n=12/group, **P<0.01,***P＜0.001, two-way ANOVA test with one factor as repeated measure for a, one-way ANOVA post hoc test for the b-e.


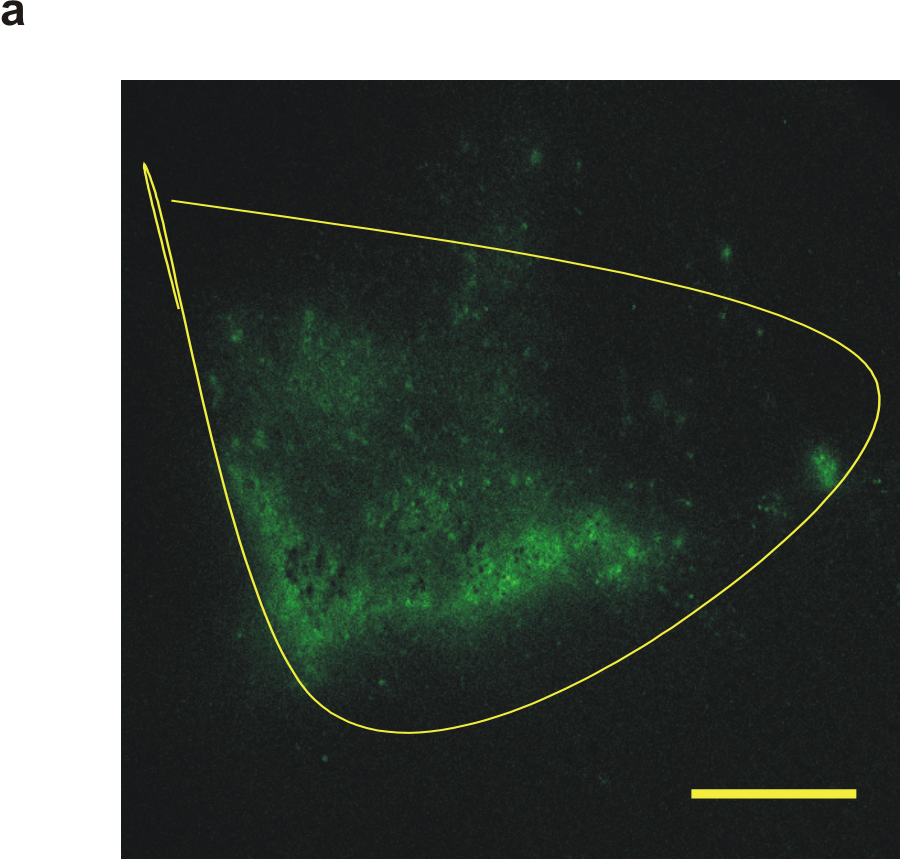


Figure S5 At two weeks after virus infusion, LV-GFP was still only localized in the BLA. Scale Bar=75 µm
